# Systematic development and adjustment of the German version of the Supportive and Palliative Care Indicators Tool (SPICT-DE)

**DOI:** 10.1186/s12904-018-0283-7

**Published:** 2018-02-17

**Authors:** Kambiz Afshar, Angelika Feichtner, Kirsty Boyd, Scott Murray, Saskia Jünger, Birgitt Wiese, Nils Schneider, Gabriele Müller-Mundt

**Affiliations:** 1 Institute for General Practice, Hannover Medical School, Carl-Neuberg-Str. 1, 30625 Hannover, Germany; 2 Paracelsus Medical University Salzburg, Strubergasse 21, 5020 Salzburg, Austria; 3 Usher Institute of Population Health Sciences and Informatics, Old Medical School, The University of Edinburgh, Teviot Place, Edinburgh, EH8 9AG UK; 4 Cologne Center for Ethics, Rights, Economics, and Social Sciences of Health, University of Cologne, Albertus-Magnus-Platz, 50923 Cologne, Germany

**Keywords:** Palliative care, General practice, Primary care, Identification tool, SPICT

## Abstract

**Background:**

The Supportive and Palliative Care Indicators tool (SPICT) supports the identification of patients with potential palliative care (PC) needs. An Austrian-German expert group translated SPICT into German (SPICT-DE) in 2014. The aim of this study was the systematic development, refinement, and testing of SPICT-DE for its application in primary care (general practice).

**Methods:**

SPICT-DE was developed by a multiprofessional research team according to the TRAPD model: translation, review, adjudication, pretesting and documentation. In a pretest, five general practitioners (GPs) rated four case vignettes of patients with different PC needs. GPs were asked to assess whether each patient might benefit from PC or not (I) based on their subjective appraisal (“usual practice”) and (II) by using SPICT-DE. After further refinement, two focus groups with 28 GPs (68% with a further qualification in PC) were conducted to test SPICT-DE. Again, participants rated two selected case vignettes (I) based on their subjective appraisal and (II) by using SPICT-DE. Afterwards, participants reflected the suitability of SPICT-DE for use in their daily practice routine within the German primary care system. Quantitative data were analysed with descriptive statistics and non-parametric tests for small samples. Qualitative data were analysed by conventional content analysis. Focus group discussion was analysed combining formal and conventional content analysis.

**Results:**

Compared to the spontaneous rating of the case vignettes based on subjective appraisal, participants in both the pretest and the focus groups considered PC more often as being beneficial for the patients described in the case vignettes when using SPICT-DE. Participants in the focus groups agreed that SPICT-DE includes all relevant indicators necessary for an adequate clinical identification of patients who might benefit from PC.

**Conclusions:**

SPICT-DE supports the identification of patients who might benefit from PC and seems suitable for routine application in general practice in Germany. The systematic development, refinement, and testing of SPICT-DE in this study was successfully completed by using a multiprofessional and participatory approach.

## Background

Prognostic uncertainty forms a major barrier to providing palliative care (PC) especially in patients with non-cancer conditions [1–5]. A crucial step to overcome this barrier is the adequate and timely identification of primary care patients with potential PC needs [6, 7]. Internationally, there are different tools supporting the identification of these patients [8–11]. The Supportive and Palliative Care Indicators tool (SPICT™) is designed to identify patients with chronic progressive diseases at risk of deteriorating and who might benefit from a palliative approach. In comparison with other identification tools, SPICT is not restricted to specific diseases and has been tested in different settings (e.g. general practice, care home, hospital) [12–15].

SPICT-DE was the first German version translated and adapted by a multidisciplinary expert group of clinicians (physicians and nurses with further qualifications in PC) from Austria and Germany in 2014/15. The development included a backward translation led by Astrid Schnabel (Germany) and Angelika Feichtner (Austria).

At the beginning of 2015, the Palliative Care Research Group of the Institute for General Practice at the Hannover Medical School conducted a first pilot study to assess the feasibility of SPICT-DE in routine clinical practice [16]. The prospective, case-control study of patients with cardiovascular or lung diseases was carried out in an internal medicine ward. The results indicated that SPICT-DE is an easy-to-use tool supporting the routine identification of patients with chronic, non-cancer conditions who might benefit from PC. Furthermore, the feedback of the participating physicians revealed a need for linguistic adaptation according to the clinical language culture and style of clinical assessment tools for physicians in Germany.

We thus aimed to further develop, refine and test SPICT-DE systematically in order to prepare it for application in general practice in German-speaking countries.

## Methods

**Fig. 1:**
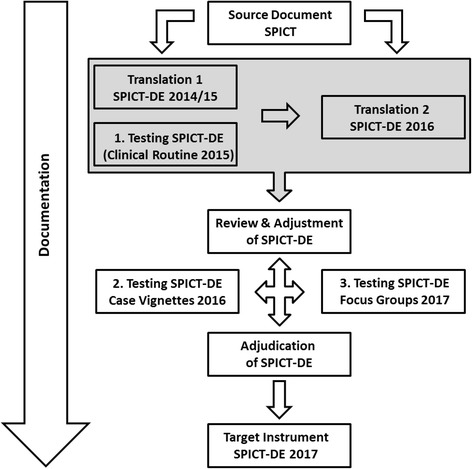
Translation and adjustment of SPICT-DE following the TRAPD model [17]

Development, refinement, and testing of SPICT-DE were conducted systematically in a multidisciplinary team (family medicine and PC, public health, psychology, sociology and nursing sciences) according to the TRAPD model [17]: translation, review, adjudication, pretesting and documentation (see Fig. 1).

### Structure of SPICT

SPICT comprises three parts: the first section with general clinical indicators (e.g. poor performance status, emergency hospital admissions or persistent symptoms), the second section for condition-specific clinical indicators (e.g. in cancer, dementia, and cardiac, pulmonary or renal diseases) and the third section with recommendations for PC actions (e.g. a conversation about deteriorating health and dying, advance care planning, a review of care goals with patients and their relatives, and referral for specialist PC). Development, structure and evaluation of the original English version of SPICT have been described elsewhere [13].

### Translation, review and adjudication of SPICT-DE

Three members of the Palliative Care Research Group of the Institute for General Practice in Hannover (KA, SJ, and GMM) reviewed and refined detailed content and language used in the SPICT-DE several times in close consultation with the SPICT research team in Edinburgh and the head of the Austrian-German expert group (AF). This work formed part of a 5 year review and consensus building approach to SPICT development, refinement and testing led by the SPICT Programme which has used the SPICT website to access the experiences and expertise of an online, international community of over 3000 clinicians and researchers. By combining an in-depth local consultation with the wider international consensus, a final agreed version of the SPICT-DE was adjudicated.

### Testing SPICT-DE

The empirical testing of SPICT-DE consisted of two stages:

1. A pretest with GPs was conducted in autumn 2016 in order to assess SPICT-DE by rating four different case vignettes. The feasibility of using SPICT-DE and the time taken to complete the rating were evaluated.
2. Two professional focus groups with GPs were conducted in spring 2017 to assess the content and layout of SPICT-DE from the GPs’ point of view. The focus groups addressed the following three goals:
  Probing the handling of SPICT-DE by rating two exemplary, selected case vignettes.
  Discussing the suitability and feasibility of SPICT-DE within the German primary care context.
  Evaluating the potential of SPICT-DE in increasing awareness of patients with potential PC needs among GPs.

### Pretest

#### Development of case vignettes

Four exemplary case vignettes (A to D) representing patients with general and/or specialist PC needs were developed. For a short description of the case vignettes see Table 1. The four case vignettes were based on qualitative longitudinal case studies reconstructed from the previous research project “ELFOP – End of life care for frail older patients in family practice” (Funding-No.: BMBF 01GY1120) conducted by the Palliative Care Research Group between 2012 and 2015 [18, 19]. All patients who took part in that project gave informed written consent prior to participation [19]. In this study, all four case vignettes were revised, modified (age, diagnoses, and layout) and anonymised before testing.

**Table 1:** Case vignettes A-D^a^

Case vignette	Main characteristics and palliative care needs	Study part
A	• 83-year-old female, widowed, living with her youngest mental handicapped son at home • Multiple chronic diseases (e.g. coronary heart disease, hypertension, morbid obesity, chronic ulcus cruris, polyarthrosis, discopathy), multiple problems in the activities of daily living, mobility-impaired • (General) palliative care indicated	• Pretest
B	• 78-year-old female, widowed, living with the family of her granddaughter at home • Multiple chronic diseases (e.g. chronic obstructive lung disease, coronary heart disease with condition after bypass surgery, cardiac arrhythmias, gastric ulcer with anemia and cachexia), advanced frailty, mobility-impaired • (General) palliative care indicated	• Pretest • Focus groups
C	• 76-year-old male, living with his spouse at home • Multiple chronic diseases (chronic heart failure, hypertension, atrial fibrillation, osteoporosis, urinary incontinence, polyneuropathy), multiple problems in the activities of daily and social living, mobility-impaired, beginning caregiver overload • Spouse affected by Parkinson’s disease • (General) palliative care indicated	• Pretest • Focus groups
D	• 65-year-old male, living with his spouse at home • Non-small-cell lung cancer with cerebellar metastases, several co-morbidities (e.g. coronary heart disease, cardiac arrhythmias, condition after stroke, chronic kidney failure, depression), multiple symptoms (e.g. pain, dyspnea, coughing, dizziness) • (Specialist) palliative care indicated	• Pretest

^a^Source: “ELFOP – End of life care for frail older patients in family practice” (Funding: BMBF 01GY1120) [18, 19]

#### Rating with and without SPICT-DE

A convenience sample of five GPs was recruited from the research practices of the Institute for General Practice for participation in the pretest. To ensure a common understanding of the term “palliative care”, a German definition based on the World Health Organisation [20] and the German Guideline “Palliative care for patients with incurable cancer” [21] was given to each participant in hard copy.

First, the participants were asked to go through the four case vignettes A to D to assess, based on their subjective appraisal, whether each patient might benefit from PC or not (“usual practice”). Then, they were asked to rate to what extent the patient’s situation described in each case vignette seemed to be clear on a 5-point-scale ranging from 1 “not clear” to 5 “very clear”.

In a second step, the participants were asked to go through each case vignette once more, this time using SPICT-DE. They were asked to indicate if the patient under consideration might benefit from PC, when reflecting on the SPICT-DE indicators. In order to monitor the indicators chosen by the raters, check boxes were added for each indicator listed in the SPICT-DE. In addition, participants were asked to indicate whether there was a change in their appraisal after using SPICT-DE, and if so, in what way. Participants were asked to state the total time in minutes needed for reviewing the case vignettes.

Review of the four case vignettes was supplemented by two questionnaires: one questionnaire with open-ended questions to judge the quality of the case vignettes and the content and language of the revised SPICT-DE, the other comprising questions about the participant’s basic sociodemographic data (e.g. sex, age, qualification in PC, current field of practice, duration of professional experiences). The paper and pencil rating of the case vignettes was completed anonymised, so that participants’ ratings of the case vignettes did not identify them individually. After analysing the results of the pretest and having refined the wording, SPICT-DE was prepared for the second phase of testing in the focus groups.

### Focus groups

Two members (KA, GMM) of the Palliative Care Research Group of the Institute for General Practice conducted the focus groups in two different quality circles: focus group A (*n* = 15) was conducted in February 2017 in Hannover (Germany) and focus group B (*n* = 13) was conducted in May 2017 in Achim (Germany). Members of the two quality circles were family physicians working in primary care practice.

After receiving information about the aims, content and format of the study, the chairs of each quality circle agreed to integrate the testing of SPICT-DE in a forthcoming meeting. All participants of the focus groups were informed that their participation would be entirely voluntary and that they would be free to withdraw from the group at any time.

A guide for moderating and structuring the focus groups was developed. Table 2 shows the timeline and content of the focus group process. After introduction and ensuring a common understanding of the term “palliative care” (see above) in a short presentation, each participant of the focus group was asked to write down individually on concept cards, what conditions and aspects would indicate that a patient is in need of PC according to their subjective appraisal and professional expertise.

**Table 2:** Timeline and content of the focus groups

Step	Action / Assessment	Time
I	Initiation • Introduction of participants and research facilitators (KA, GMM) • Introduction of the project’s objectives/aims and ensuring a common understanding of the term “palliative care” (short presentation) • Explanation of intended procedures and clarification of questions • Informed consent to participate voluntarily and to documentation of the results of the group discussion	20 min.
II	Subjective indicators • Participants were asked to write down anonymously main criteria and indicators for the identification of potential PC needs on concept cards according to their subjective appraisal	10 min.
III	Assessment without SPICT-DE (“usual practice”) • Introduction of case vignettes and explanation of case procession • Rating of the case vignettes by the participants	20 min.
IV	Assessment with SPICT-DE • Introduction of SPICT-DE and its assessment according to the SPICT guidelines (https://www.spict.org.uk) • Rating of the case vignettes by participants using SPICT-DE • Questionnaires: perception of SPICT-DE, sociodemographic data • Return of all documents anonymously in a sealed envelope • Review and clustering of concept cards by the moderators for the following group discussion simultaneously to case processing	20 min.
V	Group discussion • Participants’ feedback on the case vignettes and case processing • Participants’ feedback on impression and handling of SPICT-DE • Discussion of indicators of SPICT-DE by referring to concept cards • Final discussion of the practicability and potential benefits of SPICT-DE in the daily practice routine	40 min.
VI	Conclusion and outlook	5 min.

Participants were then asked to go through the two case vignettes B and C that had proved more difficult to categorise in the pretest phase to assess, based on their subjective appraisal, whether each patient might benefit from PC or not (“usual practice”). They were also asked to rate to what extent the patient’s situation as described in each case vignette seemed to be clear on a 5-point-scale ranging from 1 “not clear” to 5 “very clear”. After introducing SPICT-DE and its structure, participants were asked to go through the two case vignettes once more, but this time using SPCT-DE. They were asked to indicate if the patient under consideration might benefit from PC, when reflecting on the SPICT-DE indicators and whether their opinion had changed as in the pretest groups.

Feedback, contributions, and results of the group discussion which followed the individual rating exercise were documented as part of the focus group work and recorded by the research facilitators KA and GMM.

### Data analysis

As mixed methods were applied in this study, quantitative and qualitative analyses were used. Quantitative data were analysed with descriptive statistics and non-parametric tests for small samples (McNemar test and Mann-Whitney-U test). Statistical analysis was carried out with the Statistical Package for the Social Sciences (SPSS) Version 24. Open-ended questions from the supplementary questionnaire were analysed by conventional content analysis as described by Hsieh and Shannon [22]. For the focus group discussion a combination of formal and conventional content analysis was applied [22, 23].

## Results

### Pretest

#### Participants and sample characteristics

Five GPs (females *n* = 4, median age 49 years, range 39–54) participated in the pretest. They had a different degree of professional experience (median 15 years, range 4–25). One GP had additional specialist training in Palliative Medicine.

#### Rating of the case vignettes

The patients’ situations described in the four case vignettes were rated very heterogeneously by the five GPs. While case vignette A (median 4, range 1–4) and D (median 5, range 1–5) appeared to be relatively clear, the case vignettes B (median 3, range 1–5) and C (median 3, range 2–3) were considered less clear (scale ranging from 1 “not clear” to 5 “very clear”).

Before using SPICT-DE, a need for PC was identified for case A by two participants and for case C by one participant. In case vignette B and D four of the five participants shared the opinion that both patients might benefit from PC (see Table 3).

**Table 3:** Pretest rating without and with SPICT-DE (GPs, *n* = 5)

Case vignette	“Patient might benefit from palliative care”		*p*-value
	without SPICT-DE	with SPICT-DE	
	n (%)	n (%)	
A	2 (40)	2 (40)	n.s.
B	4 (80)	5 (100)	n.s.
C	1 (20)	3 (60)	n.s.
D	4 (80)	4 (80)	n.s.

McNemar-Test compared the groups; *p* < 0.05; *n.s.* not significant

With SPICT-DE to support their reviews, identification of cases with PC needs changed slightly: in case vignette B all the GPs now agreed that PC might be indicated and in case vignette C the number increased from one to three participants (see Table 3). No participant revised his/her rating for case vignette A or case vignette D.

#### Processing time

Participants were asked to note the total time needed for rating all four case vignettes. The median time for rating all the case vignettes with and without SPICT-DE was about 30 min (range 20–45). That meant an average of 7.5 min for each case vignette.

#### Feedback

Four participants evaluated the case vignettes as distinct and authentic examples. Furthermore, they made several suggestions about how to improve the wording and adapt the layout of the case vignettes. Three of five participants in the pretest group considered SPICT-DE as a useful tool to support the decision about whether a patient might benefit from PC or not. One participant remarked that a score with a cut-off would make it easier to assess SPICT-DE. Another participant noted that SPICT-DE seems to be confusing when using it for the first time, but that once familiar with the content, it became easier and clearer to use.

### Focus groups

Participants of both groups (*n* = 28) were GPs. Focus group A consisted of 15 GPs, who were members of a trans-regional geriatric quality circle. The majority of the participating GPs held a further qualification in PC (*n* = 13/15). Focus group B consisted of 13 GPs, who were members of a quality circle and a regional PC network. In contrast to focus group A, less than half of the participants in focus group B (*n* = 6/13) held a further qualification in PC. Participants in the two focus groups did not show any statistically significant differences in their sociodemographic characteristics except for median age (*p* = 0.013). Participants’ characteristics are shown in Table 4.

**Table 4:** Characteristics of participating GPs in the focus groups

	Focus group A + B	Focus group A	Focus group B
	*n* = 28	*n* = 15	*n* = 13
Sex (female); n (%)	17 (61)	10 (67)	7 (54)
Age (years); median (range)	55 (38–67)^a^	57 (42–67)^b^	45 (38–62)^c^
Further qualification in PC; n (%)	19 (68)	13 (87)	6 (46)
Professional experience (years); median (range)	24 (10–44)	29 (10–44)	17 (10–40)
Place of Work; n (%)			
Single practice	11 (39)	5 (33)	6 (46)
Group practice	16 (57)	9 (60)	7 (54)
Hospital	1 (4)	1 (7)	–

^a^*n* = 24, ^b^*n* = 13, ^c^*n* = 11

#### Assessment with and without SPICT-DE

In both focus groups, participants considered PC more often as beneficial for the patients described in the case vignettes when using SPICT-DE. With SPICT-DE, their initial subjective appraisal of the case vignettes altered. Table 5 shows the results of the case processing with and without the assessment being supported by SPICT-DE. Results of both focus groups are summarised together.

**Table 5:** Focus group rating without and with SPICT-DE

Focus group	Case vignette	“Patient might benefit from palliative care”		*p*-value
		without SPICT-DE	with SPICT-DE	
		n (%)	n (%)	
A (*n* = 15)	Case B	10 (67)	12 (80)	n.s.
	Case C	3 (20)	7 (47)	n.s.
B (*n* = 13)	Case B	9 (69)	10 (77)	n.s.
	Case C	2 (15)	8 (62)	**0.031**
A + B (*n* = 28)	Case B	19 (68)	22 (79)	n.s.
	Case C	5 (18)	15 (54)	**0.002**

McNemar-Test compared the groups; p < 0.05, significant differences in bold; n.s.: not significant

Without SPICT-DE, 19 participants (68%) saw a need for PC for the patient in case vignette B. With SPICT-DE, the number of participants who saw a need for PC increased to 22 (79%). Without SPICT-DE, five participants considered that the patient in case vignette C might benefit from PC. With SPICT-DE, the number of participants who considered that the patient might benefit from PC increased significantly to 15 (*p* = 0.002) indicating that ten participants revised their first appraisal.

Over 70% of the participants considered at least one PC action as recommended in SPICT-DE (e.g. review of current treatment and medication, communicating current and future care plan with patients and their families or support of family carers) to be indicated in the case vignettes (case B: *n* = 20/28; case C: *n* = 22/28). Over half of the participants considered as many as three PC actions to be indicated in each case vignette (median 3, range 0–5).

#### Group discussion

##### Case vignettes and rating procedures

Participants of both focus groups evaluated the case vignettes as genuine and authentic examples, reflecting patients seen routinely in general practice. On a 5-point-scale ranging from 1 “not clear” to 5 “very clear”, about two thirds of participants considered the presented patient scenario in each case vignette as clear or even very clear (case vignette B: median 4, range 2–5; case vignette C: median 4, range 1–5). Participants stated that the indicators of the SPICT-DE are more detailed and broadly defined than the subjective criteria. Especially in ambiguous cases the structured presentation of these objective criteria for PC needs led to an alteration of the subjective appraisal.

##### Quality and feasibility of SPICT-DE

Participants of both focus groups agreed that SPICT-DE covers relevant indicators to identify patients who might benefit from PC. Furthermore, SPICT-DE comprises important recommendations in providing adequate and timely PC.

By comparing the participants’ individually generated indicators with the indicators in SPICT-DE, we saw that two sets of indicators were almost identical and differed only in that the indicators written down on the concept cards were more detailed and concrete. Group discussion confirmed that the indicators included in SPICT-DE have good face validity for practicing GPs in Germany and are sufficiently detailed and clear to use to support patient identification and prompt actions for the most important and critical aspects of the provision of PC. Participants considered the combination of general and clinical indicators with specific recommendations for PC actions of the SPICT-DE to be especially helpful in supporting improved provision of PC for people with non-cancer illnesses, where the timely identification and decision-making is often more difficult.

##### Using SPICT-DE in routine clinical practice

In focus group A, some of the participants said that with SPICT-DE the number of patients identified in general practice that might benefit from PC would increase significantly. They shared the opinion that a considerable proportion of their population of older patients with multimorbidity could benefit from PC according to SPICT-DE. Other participants agreed that SPICT-DE could be helpful in deciding whether a code for generalist PC according to the German remuneration system (Einheitlicher Bewertungsmaßstab, EBM) would be indicated or not. When asked to give an opinion via the anonymised questionnaires, all the participants in group A and all but two in group B were enthusiastic about SPICT-DE and could imagine using it as part of their daily practice routine to support them in identifying patients who might benefit from general and/or specialist PC. One participant, who could not imagine using SPICT-DE in daily practice routine, stated that the expression “tool” is confusing and that the SPICT-DE is nothing but a list of indicators. Furthermore, this participant was convinced that the gut feeling is an important aspect itself which cannot be replaced by indicators. Another participant was undecided and stated that SPICT-DE might be too complex for daily practice routine. Table 6 shows the answers of participants in focus group B concerning the possible further use of SPICT-DE in routine clinical practice.

**Table 6:** Use of SPICT-DE in daily practice routine (Focus group B)

Code	Use of SPICT-DE^a^	Explanation
QZB 01	Yes	“Important indicators, which might support the decision-making, especially when added together”
QZB 02	Yes	“Supports structured medical history”
QZB 03	Yes	“It increased the awareness for advance care planning”
QZB 04	No	“For me, the expression “Tool” is misleading; it is simply a list of indicators. I have already used some of these indicators, whatever remains is the gut feeling”
QZB 05	Yes	“So far, decision-making was based on my intuition – a decision-making based on objective criteria is a reasonable supplement”
QZB 06	Yes	“It increases the awareness”
QZB 07	Yes	“Reasonable decision aid, especially for case conferences”
QZB 08	Yes	“A helpful tool, especially for ambiguous cases”
QZB 09	Yes	“It is helpful in cases of uncertainty”
QZB 10	Yes	“Supports the decision-making and seeing the big picture”
QZB 11	Yes	–
QZB 12	Yes	–
QZB 13	Undecided	“I don’t know yet, seems to be complex in parts”

^a^Question: “Could you imagine using SPICT-DE in your daily practice routine? Please justify your answer”

## Discussion

In this research paper, we report the results of the first pilot study on developing and testing the German version of SPICT: (1) a pretest with five GPs rating case vignettes with and without SPICT and (2) focus groups with 28 GPs to further evaluate and discuss SPICT-DE. The revision and refinement of SPICT-DE was made according to the TRAPD model and in close consultation with the SPICT research team in Edinburgh and the head of the Austrian-German expert group. These results provided the basis for the final multidisciplinary version of the SPICT-DE 2017, which can be downloaded free of charge from the SPICT website (http://www.spict.org.uk).

Participants of the focus groups shared the opinion that SPICT-DE has a great potential to increase awareness for end-of-life issues and support the provision of advance care planning. This is remarkable as almost two thirds of participating GPs had a further qualification in PC. Our results promote that especially in ambiguous cases SPICT-DE might support the identification and decision-making. There was a significant alteration in the rating of case vignette C when using SPICT-DE, especially in focus group B where only 46% of the participants had a further qualification in PC. It can be assumed that using SPICT-DE might be even more beneficial for those GPs who have no further qualifications in PC.

There is a controversy in the international literature about the use of a cut-off value in SPICT. Some authors have considered whether SPICT could be used as a tool to determine the prognosis of patients with PC needs and therefore advocated for the necessity of having a cut-off value in SPICT [15]. However, the current versions of SPICT 2017 and SPICT-DE 2017 do not include any cut-off value, in line with increasing recognition that prognostication for individual people is unreliable and unhelpful in promoting earlier PC [24]. Identifying people at risk of deteriorating health on the basis of their clinical situation using SPICT facilitates timely identification of a broader group of patients who might benefit from PC. Indeed, our study showed that without a cut-off value, using SPICT-DE led to an increased number of patients identified with PC needs. In this context, concerns about potential harm of a cut-off score need to be reflected in two respects. On the one hand, there may be worries about deprivation from curative and healing opportunities among patients meeting the cut-off score. On the other hand, patients not meeting the cut-off score may be withheld opportunities of timely addressing end-of-life issues, including advance care planning. The identification of these patients is the prerequisite for initiating a suitable PC programme, which may involve a critical review of their medication to avoid polypharmacy and treatments no longer of benefit, a conversation on end-of-life issues, advance care planning, a review of care goals with the patients and their relatives, or simply a prompt to reassess the patient using SPICT at a later date. The identification process should be followed by a careful assessment of actual PC needs in the identified patient.

Our results indicate that the application of SPICT-DE is feasible in daily clinical practice. The time needed to apply SPICT-DE in general practice would be less than in a testing situation, as GPs usually know their patients better and over time would become more familiar with the SPICT-DE indicators so able to use them more rapidly to support patient identification.

### Strengths and limitations

The systematic development, refinement, and testing of SPICT-DE in this study was successfully completed by using a multiprofessional and participatory approach. In particular, the involvement of GPs as target group of potential users in the development process was an important consideration to increase acceptance of SPICT-DE before its implementation. A participatory research approach to refine and develop SPICT has been adopted from the outset. This approach seems particularly suited for the development of a descriptive tool designed for use in all care settings and in many different countries [13].

According to Johanson and Brooks a sample size of about 30 participants is considered adequate for pilot studies aiming at scale or tool development [25]. With 28 participants the sample size in this pilot study is close to these recommendations [25, 26]. A limitation is the application of case vignettes for the testing of SPICT-DE. In daily practice the use of the SPICT-DE may be different. However, the selected case vignettes were considered as realistic and representative. Another limitation is the gender imbalance in the focus groups as almost two thirds of participating GPs were females. Although this mismatch was not statistically significant and did not affect the rating results, potential gender-specific aspects in the focus group discussions have to be taken into account.

## Conclusions

SPICT-DE is a helpful and practical tool to support the identification of patients who might benefit from PC. The introduction of SPICT-DE in the general practice setting in Germany might change the usual identification strategy and possibly increase GP’s awareness in providing PC for patients with different chronic progressive diseases. The tool may contribute to consolidating skills and competencies of GPs in identifying patients with potential PC needs and increase their confidence in initiating PC. The results may also contribute to the overall improvement and optimisation of primary PC by GPs in Germany and German-speaking countries. Further research should evaluate the implementation of SPICT-DE in routine daily practice.

## Abbreviations

GP: General practitioner
PC: Palliative care
SPICT: Supportive and Palliative Care Indicators Tool
TRAPD: Translation, review, adjudication, pretesting and documentation
